# Explosive detonation causes an increase in soil porosity leading to increased TNT transformation

**DOI:** 10.1371/journal.pone.0189177

**Published:** 2017-12-27

**Authors:** Holly A. Yu, Niamh Nic Daeid, Lorna A. Dawson, David A. DeTata, Simon W. Lewis

**Affiliations:** 1 Department of Chemistry, Curtin University, Perth, WA, Australia; 2 Curtin Institute of Functional Molecules and Interfaces, Curtin University, Perth, WA, Australia; 3 Centre for Anatomy and Human Identification, School of Science and Engineering, University of Dundee, Dundee, United Kingdom; 4 The James Hutton Institute, Aberdeen, Scotland, United Kingdom; 5 Forensic Science Laboratory, ChemCentre, Perth, WA, Australia; The University of Sydney, AUSTRALIA

## Abstract

Explosives are a common soil contaminant at a range of sites, including explosives manufacturing plants and areas associated with landmine detonations. As many explosives are toxic and may cause adverse environmental effects, a large body of research has targeted the remediation of explosives residues in soil. Studies in this area have largely involved spiking ‘pristine’ soils using explosives solutions. Here we investigate the fate of explosives present in soils following an actual detonation process and compare this to the fate of explosives spiked into ‘pristine’ undetonated soils. We also assess the effects of the detonations on the physical properties of the soils. Our scanning electron microscopy analyses reveal that detonations result in newly-fractured planes within the soil aggregates, and novel micro Computed Tomography analyses of the soils reveal, for the first time, the effect of the detonations on the internal architecture of the soils. We demonstrate that detonations cause an increase in soil porosity, and this correlates to an increased rate of TNT transformation and loss within the detonated soils, compared to spiked pristine soils. We propose that this increased TNT transformation is due to an increased bioavailability of the TNT within the now more porous post-detonation soils, making the TNT more easily accessible by soil-borne bacteria for potential biodegradation. This new discovery potentially exposes novel remediation methods for explosive contaminated soils where actual detonation of the soil significantly promotes subsequent TNT degradation. This work also suggests previously unexplored ramifications associated with high energy soil disruption.

## Introduction

Explosives, such as 2,4,6-trinitrotoluene (TNT), may be present as contamination in soils at various locations, such as at former military training sites, explosives manufacturing sites or sites of bombing incidents [1–5]. As explosives may be toxic and harmful to the environment [1, 6–11], it is important to remediate these contaminated brownfield sites before regeneration and redevelopment can safely occur [6, 9, 12–18]. A significant body of research has been performed to investigate the bioremediation of explosives-contaminated soils [5, 8, 19–38], however much of this has assessed ‘pristine’ soils, unexposed to an actual detonation process.

Recognising that a detonation is likely to affect the structure of a soil, more recent research has focused on so-called ‘fractured’ soils [31, 32]. In some cases, analysis by scanning electron microscopy (SEM) has been performed to look at the effects of a detonation on the exterior of soil aggregates [32]. However, no previous research has taken this a stage further to assess the direct effect of a detonation on the internal structure of soils, particularly with regards to how this may affect the fate of any explosives within the soils. Our current work is therefore the first of its kind to investigate the effect of a detonation on the internal structure of a soil, knowledge which may aid the bioremediation of explosives in brownfield sites as well as provide insight into the internal disruption of soil from high energy insult.

## Materials and methods

### Preliminary detonation trials

Preliminary detonation trials were performed to determine the most suitable charge position for later experiments and to assess explosives loadings onto soil following a detonation. Throughout this work, all detonations were performed in conjunction with the Western Australia Police Tactical Response Group–Bomb Response Unit. Permission was granted to the Unit for the detonations to be performed at Orange Grove Shooting Association, Gosnells, Western Australia and Wooroloo Prison Farm, Wooroloo, Western Australia. 2 kg soil samples of three dried, sieved soils (landscape, native and Spearwood) were weighed out into large 4.5 L plastic screw-cap bottles. Spearwood soil was kindly donated by the Soil Laboratory of ChemCentre, Western Australia. Native and landscape soils were purchased from Soils Ain’t Soils, Perth, Western Australia. Two such bottles were prepared for each of the three soils. Properties of the soils are provided in S1 Table. 3 days prior to the detonations, 100 mL tap water was added to each bottle (with the mass of spiked water equalling 5% of the total soil mass). Each bottle was then manually tumbled for approximately 5 minutes until the water was dispersed evenly throughout the soil. The soils were stored at room temperature for 3 days prior to the detonations. For these initial detonations, two conditions were trialled: detonations performed over soil, or detonations performed in contact with soil. The procedures for the two conditions are outlined below.

#### Detonations over soil

A new tarpaulin was placed on the ground, and a 2 x 2 m piece of black plastic film laid over it. A 1 x 1 m square was marked in the centre of the black plastic using 1 m lengths of string. The tarpaulin and black plastic film were then secured to the ground using heavy-duty tape. The pre-wetted soil was sprinkled evenly across the black plastic film, ensuring the soil remained within the pre-marked 1 x 1 m area. Using star pickets and string, a 25 g booster connected to a detonator was suspended 50 cm above the centre of the square of soil, before the charge was detonated. It was ensured that the booster was placed perpendicular to the plane of the layer of soil. Following detonation, the post-blast soil was poured back into its original plastic tub prior to transportation to the laboratory for analysis.

#### Detonations in contact with soil

A new tarpaulin was placed on the ground, and a 2 x 2 m piece of black plastic film laid over it. The tarpaulin and black plastic film were then secured to the ground using heavy duty tape. A 55 L metal bin was placed in the centre of the black plastic film, and an additional piece of black plastic film was used to line the bin. Following this, the soil was placed at the bottom of the bin, and the charge, connected to a detonator, inserted approximately 3 cm into the soil. The charge was detonated, and the soil was poured back into its original tub ready for transportation to the laboratory for analysis. The bin lids were not used during the detonations.

Following the first such detonation of this type, a slight modification was made for the subsequent two detonations of this type. The bin was 2/3 filled with builder’s bricks and then lined with black plastic film, before adding the soil, in order to position the soil closer to the top of the bin and reduce the confinement of the booster. In the first detonation with the greatest confinement, confining the soil and charge in this manner increased the explosive effect which expelled the majority of the soil from the scene, resulting in low soil recoveries. For the following two detonations, because the confinement was reduced with the soil placed closer to the top of the bin, this reduced the resulting blast effect and enabled a higher proportion of soil to be recovered for analysis.

#### Sample processing after preliminary detonation trials

When the post-blast soil was received in the laboratory, each container of soil was weighed, to determine the percentage of soil recovered from each blast. This was referenced with respect to the dry mass of the starting soils, prior to wetting, as it was apparent during the detonations that the water content of the soils had changed during the trials. Due to the high ambient temperature during the detonation trials (approximately 33°C), it is estimated that close to total evaporation of the water had occurred. 3 approximately 40 g samples of each soil were portioned off using the cone and quarter method for later size fraction analysis. Following this, each container was thoroughly manually tumbled for 10 minutes to ensure the post-blast soil samples were homogenised prior to analysis.

6 x 5 g (±0.05 g) soil samples were taken from each of the different detonation conditions. 1.25 mL water was added to each vial, to give a water content equal to that used in our previous solution-spiked soil [39]. It should be noted that even though the soils had been pre-wetted prior to the range day, the soils dried out during their time spent on the black plastic, which heated up significantly during the detonations, and during exposure to the heat of the detonations. Following this, each soil sample was extracted and analysed to determine the levels of explosives present and their degree of homogeneity within the bulk soil sample. Extraction was performed as follows: 5 mL MeCN was added to each vial, and the vials sonicated for 30 min, before filtering the resulting extracts through 0.2 μM syringe filters and placing 625 μL of this filtered extract into an LC vial. 355 μL Milli-Q water was added, along with 20 μL of 0.01 mg/mL 1,2-dinitrobenzene in acetonitrile, to give a final volume of 1000 μL. 50 μL of each such sample was injected in duplicate onto the LC, and the results averaged.

Additionally, in order to assess whether any of the detonations had given rise to soil fracturing, a sample was taken from each of the bulk post-blast soil samples and analysed using Scanning Electron Microscopy (SEM). Instrumental procedures and parameters for these SEM studies are outlined in more detail below.

### Final detonation conditions for ageing studies

Setup was performed by adapting the conditions used in the preliminary soil detonation trials. 1 kg samples of the dried, sieved soils were weighed into 4.5 L plastic screw-cap bottles. 3 days prior to the detonations, 50 mL tap water was added to each bottle (with the mass of spiked water equalling 5% of the total soil mass). Each bottle was then manually tumbled for approximately 5 minutes until the water was dispersed evenly throughout the soil. The soils were stored at room temperature for 3 days prior to the detonations.

All detonations were over a layer of soil, based on the procedure used for the earlier detonations. However, in order to increase the loading of explosives residues onto the soils, three sequential detonations, each using one booster, were performed over each soil. Following the first and second detonations in each set of three detonations, any soil which had been displaced outside the pre-marked 1 x 1 m square, but remained on the black plastic film, was shaken back into the pre-marked square, prior to the next detonation, to ensure that the soil was directly beneath the booster during the detonation.

#### Sample processing after final set of detonations

Following receipt into the laboratory, each bulk soil sample was held in a freezer at -20°C prior to portioning off into aliquots (with this entire process completed within two hours of receipt of the soils into the laboratory). Note that in this work, the effect of freezing on soil structure was not examined—this would be interesting to explore in future work. Each soil was passed through a 2 mm sieve, to remove any extraneous material not present prior to the detonations (mostly consisting of dried plant matter from the detonation site, likely due to the negative pressure phase from during detonation). As each soil was initially passed through a 2 mm sieve prior to the detonations, this process was not thought to have an effect on subsequent size fraction analyses. The bulk soil samples were then weighed, to determine the percentage soil recovery, before 3 approximately 40 g samples of each soil were portioned off using the cone and quarter method for later size fraction analysis.

The remaining soil was poured into a disposable aluminium tray and mixed thoroughly for 10 minutes using a disposable plastic spoon, until the soil appeared to be thoroughly homogenised. A set of 6 x 5 g (±0.05 g) samples was then portioned off in order to quantify the levels of TNT and PETN which had been deposited onto each of the soils, following the method described for the preliminary detonation trials. In order to quantify the explosives loadings onto the three soils, each set of 6 samples designated for quantitation for each soil were extracted as for the samples from the preliminary detonation trials. Analysis by HPLC-DAD revealed that the levels of TNT and PETN in the Spearwood and landscape soil samples were falling within the limits of the calibration range prepared for this work, so extraction and analysis was performed in the same manner as for samples from the preliminary detonation trials. The native soil had significantly higher levels of TNT and PETN (approximately 100 times higher than those in the Spearwood and landscape soils), which caused the peak areas to fall outside the calibration range. Following extraction and filtration of the native soil extracts, native soil samples were therefore diluted 1:10 compared to the Spearwood and landscape soil extracts, and an injection volume of only 5 μL was used, compared to the 50 μL injection volume used for the Spearwood and landscape soils. This alteration brought the TNT and PETN peak areas from these native soil samples back to within the prepared calibration range.

### Soil size fraction determination

Size fractions of the pre- and post-blast soils were determined as follows: The cone and quarter method [40] was used to produce 3 aliquots of each pre- and post-blast soil (each at least 40 g). After drying overnight in an oven at 60°C, each aliquot of soil was passed through progressively finer sieves (>1 mm, 0.495–1 mm, 0.250–0.495 mm and <0.250 mm), to determine the percentage by mass of each size fraction of the soils. Details of the sieves used are as follows: 250 μm sieve: mesh no. 60, aperture 250 μm, Endecotts Test Sieves Ltd, London, England; 495 μm sieve: mesh no. 32, The Tyler Standard Screen Scale, The W.S. Tyler company, Cleveland, Ohio; 1000 μm sieve: Mesh no. 16, The Tyler Standard Screen Scale, The W.S. Tyler Company, Cleveland, Ohio. For size fraction determination of the detonations where the booster was in contact with soil at the time of detonation, any large pieces of black plastic or brick resulting from the experimental setup were manually removed before weighing. These size fraction analyses were performed by sieving triplicate soil samples through a tower of progressively finer sieves, and determining the proportion of each size fraction (by mass).

### SEM analyses of soils

Preliminary SEM analyses were performed on an Obducat CamScan 3200LV SEM using Helios software. Secondary electron imaging (SEI) and Backscattered electron imaging (BEI) images were captured, using a scan speed of S4, gun voltage of 25.00 kV and emission of 92 μA. For further SEM analyses, sample preparation was performed by rinsing a small amount of soil with distilled water (to remove any sediment coating the outside of the soil grains) and then ethanol, before oven-drying the samples for 10 min at 40°C. The cleaned soils were then transferred onto an SEM stub and sputter coated using a thin layer of platinum prior to analysis. This set of SEM analyses was performed on a Carl Zeiss Sigma VP Field Emission Gun Scanning Electron Microscope coupled to a Bruker Quantax 400 Energy Dispersive Spectrometer equipped with an Xflash 5030 Silicon Drift Detector. Analysis was carried out using an accelerating voltage of 20kV in variable pressure mode, with a mix of 40% Secondary Electron and 60% Backscattered Electron.

### Preparation of post-blast samples for ageing studies

Following the final set of detonations, samples were portioned off for ageing studies: 36 x 5 g (± 0.05 g) samples were portioned off into 50 mL amber glass bottles for each of the detonations. 1.25 mL water was added to each vial, to give a water content equal to that of our comparative solution-spiked soil samples [39]. Although water was initially added to the soils 3 days prior to the detonations, this water appeared to undergo complete evaporation during its exposure to the warm Western Australian climate during the detonation set-up and detonations, so this water (equivalent to 0.25 mL per 5 g soil sample) was re-introduced into each post-blast soil sample, along with a further 1 mL water to represent the 1 mL aqueous spiking solution introduced for each of the in-lab spiked samples [39]. For each soil, 12 samples were stored at room temperature, 12 refrigerated at 1°C and 12 frozen at -20°C, with two samples from each soil analysed at time points of 1, 4, 7, 14, 28 and 42 days. Samples were extracted on their designated extraction day, with the majority of LC analyses performed on the day of extraction.

For the landscape and Spearwood soils, extraction was performed exactly as for the preliminary detonation trial samples, detailed earlier. For the native soil, due to higher explosives loadings being present (detailed later), following extraction, samples were diluted 1:10 compared to the Spearwood and landscape soils, and an injection volume of only 5 μL was used, compared to the 50 μL injection volume used for the Spearwood and landscape soils. This ensured the peak areas of TNT and PETN from the native soil samples fell within the calibration curve limits used throughout this work.

### μCT analyses

#### Sample preparation

Intact, dried soil aggregates (1–2 mm) were selected from each of the 9 conditions (landscape, native and Spearwood soils, either pre-blast, post-blast detonated in contact with soil, or post-blast detonated above soil), with a total of three aggregates analysed from each condition.

The aggregates were prepared in one of two ways prior to CT scanning. In the first method, a small piece of double-sided sticky tape was placed over the tip of a plastic micropipette tip and smoothed into place. The tape was then gently touched to the chosen aggregate, to mount the aggregate onto the pipette tip. The base of the pipette tip was then mounted vertically into a metal sample stage clip, prior to insertion into the scanner. In later scans, a folded piece of foam was inserted into the clip beneath the pipette tip, to provide some friction and minimise any potential slippage of the pipette tip throughout the 360° rotation required for the scans.

In the second method, individual aggregates were gently placed into the hollow centre of a micropipette tip and the pipette tip tilted to become vertical, to allow the aggregate to naturally fall and be held in place by the angled walls of the pipette tip. Prior to mounting into the sample stage clip, the pipette tip was inserted into an inverted pipette tip to provide sufficient height for the clip to avoid hitting the X-ray source during its rotation. Images of the two set ups are provided in the Supporting Information in S1 Fig.

#### μCT data acquisition

Scanning was performed on a Nikon XT H 225 ST CT scanner, auto-conditioned daily. The CT scanner uses Nikon Metrology X-TEK Inspect-X Version XT 4.3.1. Briefly, the scanning procedure involved: Placing the sample holder into the stage and securing in place. The aggregate was then located on the screen and magnified such that upon a 360° rotation of the sample, the aggregate edges spanned approximately 90% of the screen. The Image Optimisation parameters were adjusted manually to improve the contrast of the aggregate on the screen. Typical parameters used include: Beam energy 150 kV; Beam current 193 μA; Power 29 W; Exposure 2 f.p.s.; Gain 18 XdB. Following this, a new shading correction was created prior to the scan. Each scan was acquired using 4 frames per projection, and mostly 1440 (occasionally 720) projections, to give a total acquisition time of 48 (occasionally 24) minutes. No X-ray filters were applied during acquisition. Details of subsequent image reconstruction, thresholding, volume analyses and porosity determination are provided in the Supporting Information.

## Results and discussion

Particle size fraction analyses of the pre- and post-blast soils revealed a tendency of the soils to shift to a smaller average particle size following detonations, an effect which was more pronounced when the explosive charge was detonated in contact with the soils, rather than above the soils (S2 Fig). As smaller particles will have a larger surface area to volume ratio, the data from S2 Fig suggests that the surface areas of the landscape and native soils in particular increased following each of the detonations.

**Fig 1:**
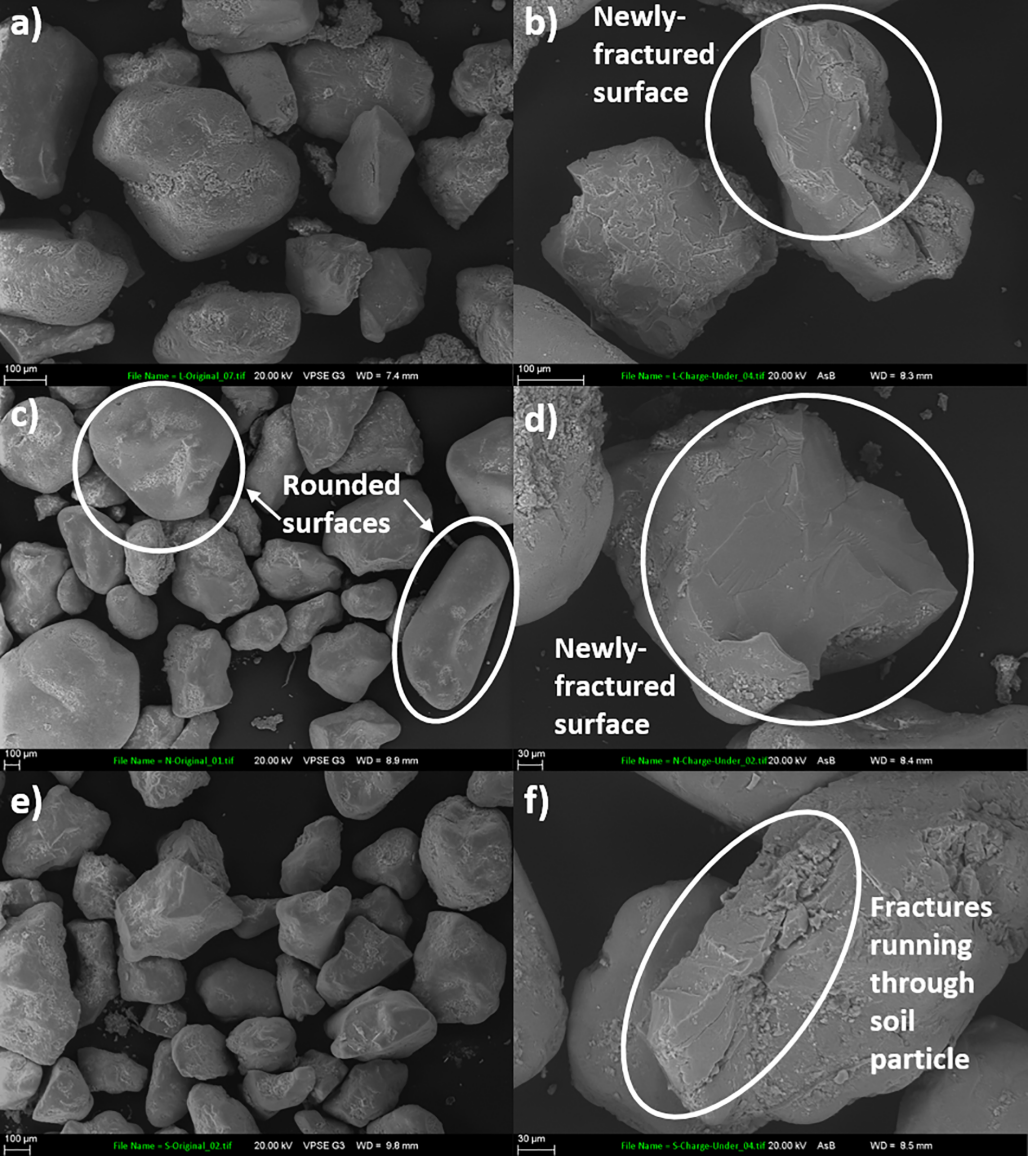
SEM images of a) pre-blast landscape soil, b) post-blast landscape soil, c) pre-blast native soil, d) post-blast native soil, e) pre-blast Spearwood soil and f) post-blast Spearwood soil. In each of the post-blast soils illustrated in this figure, the explosive was detonated in contact with the soils. Newly-cleaved planes and fractures are visible within each post-blast soil. Note that the post-blast soils are displayed at a higher magnification than the pre-blast soils, to more clearly illustrate the detonation-induced damage to the soils. Source: Evelyne Delbos, James Hutton Institute.

SEM analysis of the post-blast soils revealed the presence of newly-fractured surfaces. Example SEM images are presented in Fig 1, with enlarged comparative pre-blast SEM images of the three soils provided in S1–S3 Figs. Our SEM and size fraction analyses provide valuable insight into the effects of a detonation on the exteriors of soil particles, compared to pre-blast, ‘pristine’ soils.

Following this, we performed micro Computed Tomography (μCT) analyses to investigate what effect the detonations had on the internal structure of the soil aggregates, something which has, to our knowledge, never been explored before for explosive detonation. μCT scanning is an emerging technique for the analysis of soil structure [41–44] and is capable of providing information on the internal structure of soil aggregates, such as pore distribution and connectivity [43–45].

**Fig 2:**
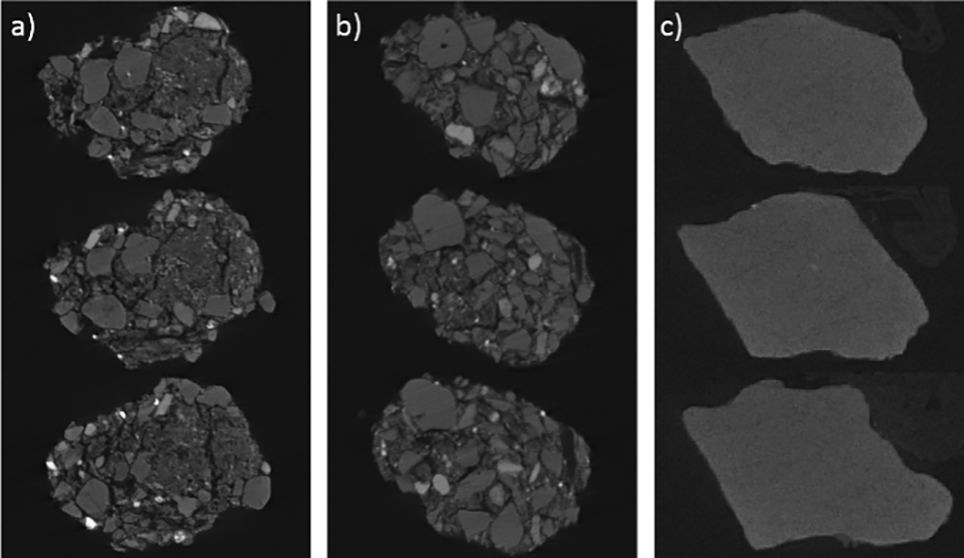
μCT slices taken from a) a pre-blast landscape soil aggregate; b) a pre-blast native soil aggregate; and c) a pre-blast Spearwood sand grain. All aggregates measured 1–2 mm in diameter. Source: Holly Yu, Curtin University/University of Dundee.

Such visualisations can therefore provide insight into the micro-scale habitat of microorganisms such as bacteria in soil [43, 44], and may enable connectivity and pathways to be inferred about the mode of transport and operation of these bacteria. For example, Akbari et al. [44] assessed the role of soil pore size distribution on the biodegradation of hexadecane in soils. The authors characterised the pore size and pore distribution of the aggregates using μCT and N_2_ adsorption techniques, and also assessed the ability of hexadecane-degrading bacteria to pass through different pore sizes, finding that hexadecane biodegradation only occurred if pores were of a bioaccessible size (i.e 5 μm or larger). When we analysed our three soil types using μCT scanning, we observed large differences between the internal structures of the soils, with two soils (landscape and native) having similar, aggregated, interiors, whereas the Spearwood sand appeared to be formed of homogeneous pieces, with limited internal definition (Fig 2).

**Fig 3:**
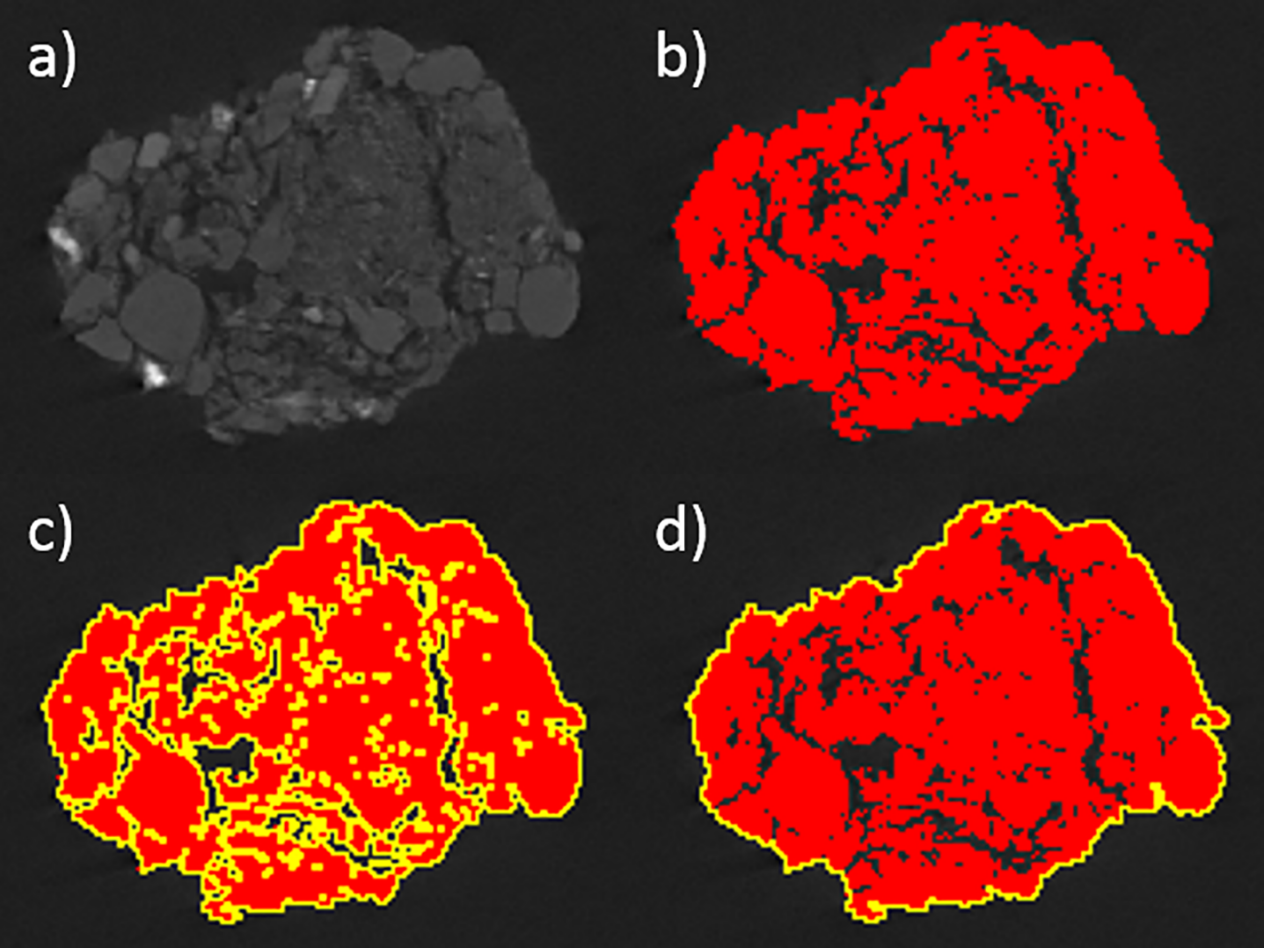
Images of a landscape aggregate slice, showing a) the original slice; b) the slice after thresholding; c) the thresholded slice following the 'Create Selection' command; and d) the thresholded aggregate slice with solely its external edge selected.

The percentage porosity of the different aggregates was calculated and the porosities of our pre- and post-blast soils compared. Example figures from this porosity determination sequence are provided in Fig 3; this process is detailed fully in the Supporting Information.

**Fig 4:**
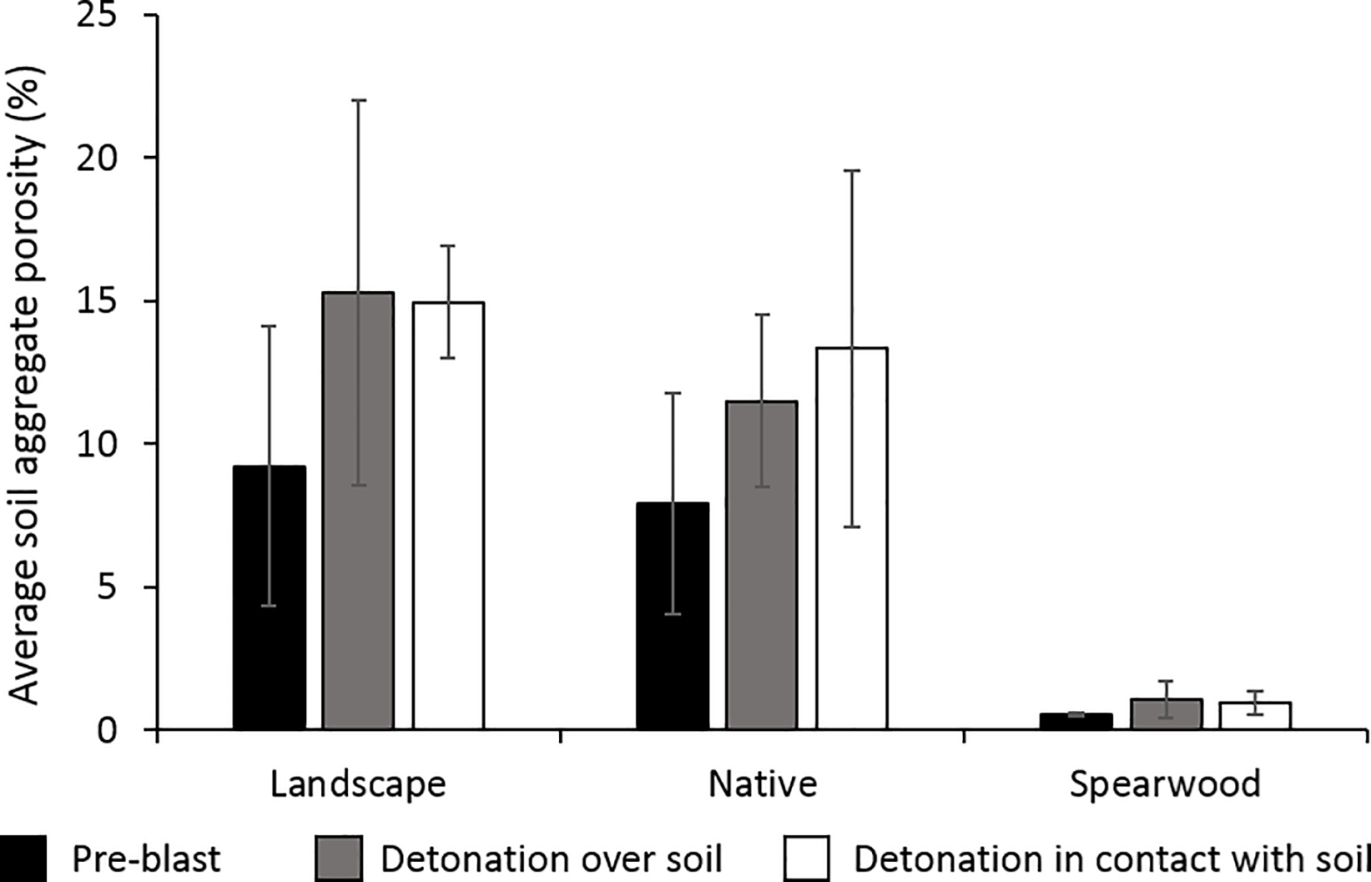
Calculated average porosities from the pre- and post-blast landscape, native and Spearwood soil aggregates. Error bars show standard deviations between three measurements.

From our porosity calculations we determined that the process of a detonation led to an increase in the overall porosity of the aggregates in all three soils (Fig 4). This information is also tabulated in the Supporting Information, S2 Table.

**Fig 5:**
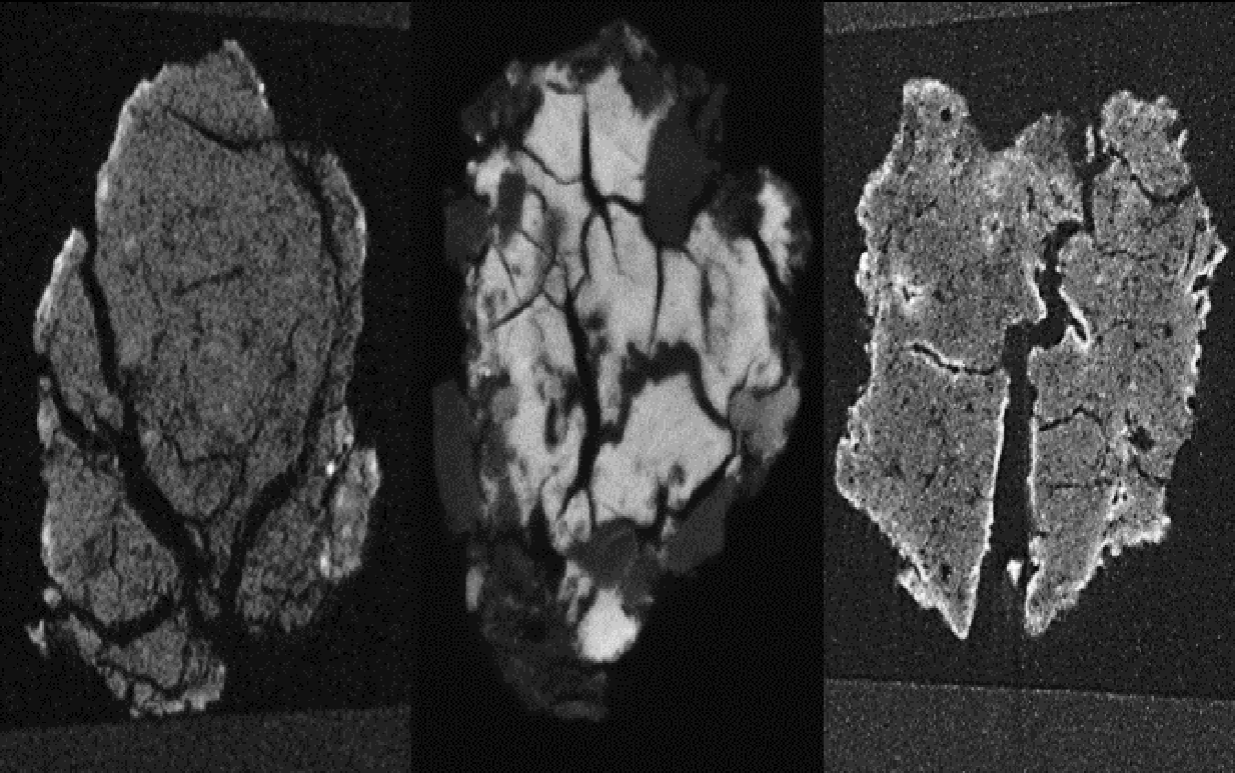
Images taken from post-blast aggregates displaying large cracks throughout the aggregate structures (left to right: a native soil aggregate following explosive charge detonation in contact with soil; a native soil aggregate following explosive charge detonation over soil; a landscape soil aggregate following explosive charge detonation over soil). Aggregate size 1–2 mm. Source: Holly Yu, Curtin University/University of Dundee.

By comparing the average pre- and post-blast porosities displayed in Fig 4, it can clearly be seen that the process of a detonation has given rise to a higher porosity within the analysed aggregates. In addition, μCT-scanning revealed several visible ‘cracks’ within some of the aggregates (Fig 5).

In parallel to these analyses, we also monitored the degradation of TNT using HPLC over a 6-week timeframe in the soils where our explosive charge was detonated over our three different soils, comparing this degradation to our previous results [39] where TNT was spiked into the same three soil types using a solution of explosives. In the solution-spiked landscape soil samples, the TNT was lost gradually over the 6-week timespan of this study [39]. In contrast, in the solution-spiked Spearwood samples, the majority of the TNT spiked into the samples was lost within the first day following sample spiking; at the same time, the rapid emergence of a TNT microbial degradation product (4-amino-2,6-dinitrotoluene; 4-ADNT) was revealed.

**Fig 6:**
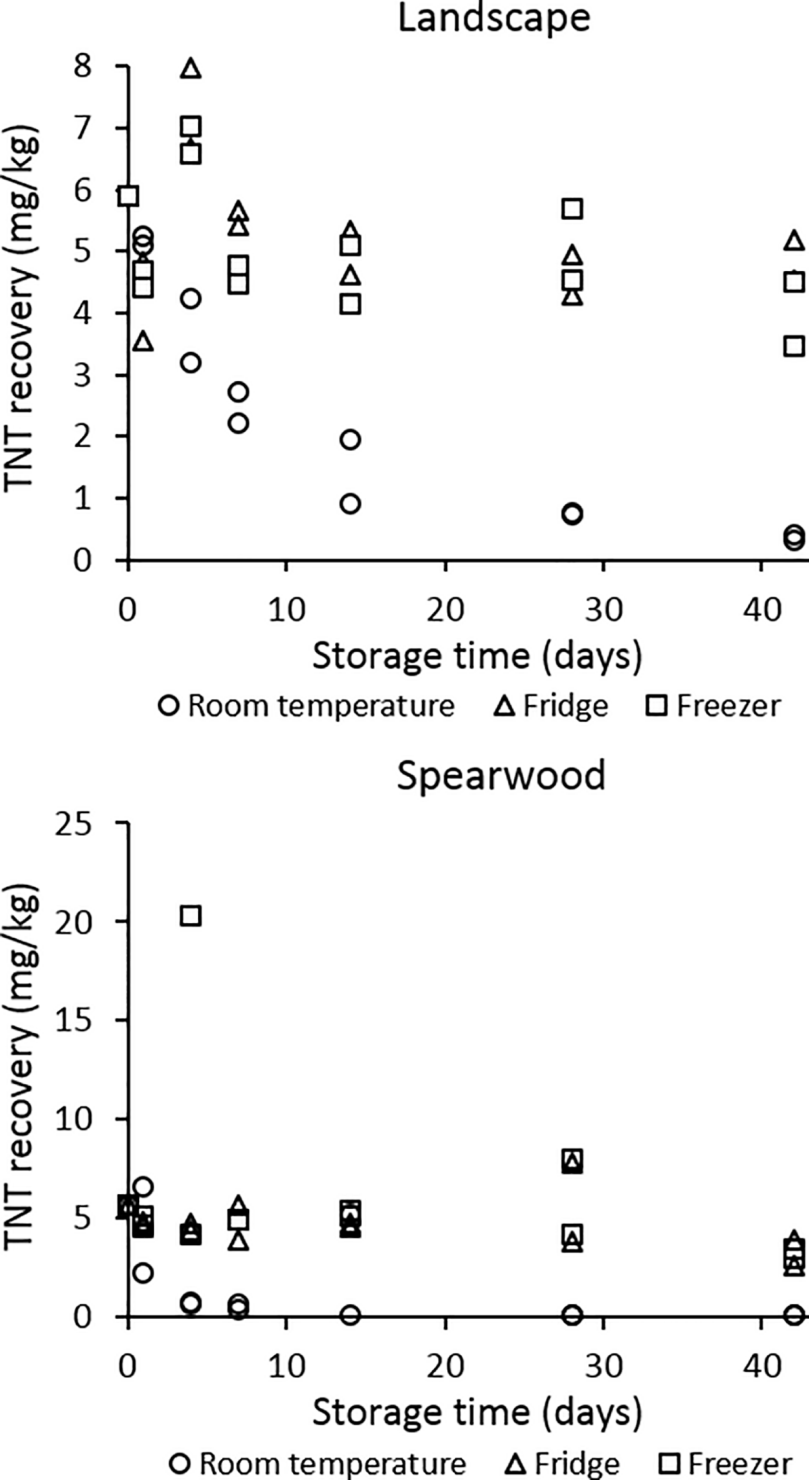
TNT recoveries obtained from post-blast landscape and Spearwood soil samples and stored at 3 temperatures, extracting over 6 weeks.

Fig 6 displays TNT recoveries obtained from this work’s comparative post-blast landscape and Spearwood soil samples and stored at 3 temperatures, extracting over 6 weeks.

In this work the rate of TNT loss from solution-spiked [39] and detonated soils was compared. In our solution-spiked soils [39], TNT was spiked at a level of approximately 2 mg/kg of soil. In our detonated soils, a slightly higher quantity of TNT was spiked into the soils, owing to the limited control over the detonation process. For this reason, simply superimposing the solution-spiked and detonation-based recoveries may not enable a thorough comparison to be made between the degradation rates.

**Fig 7:**
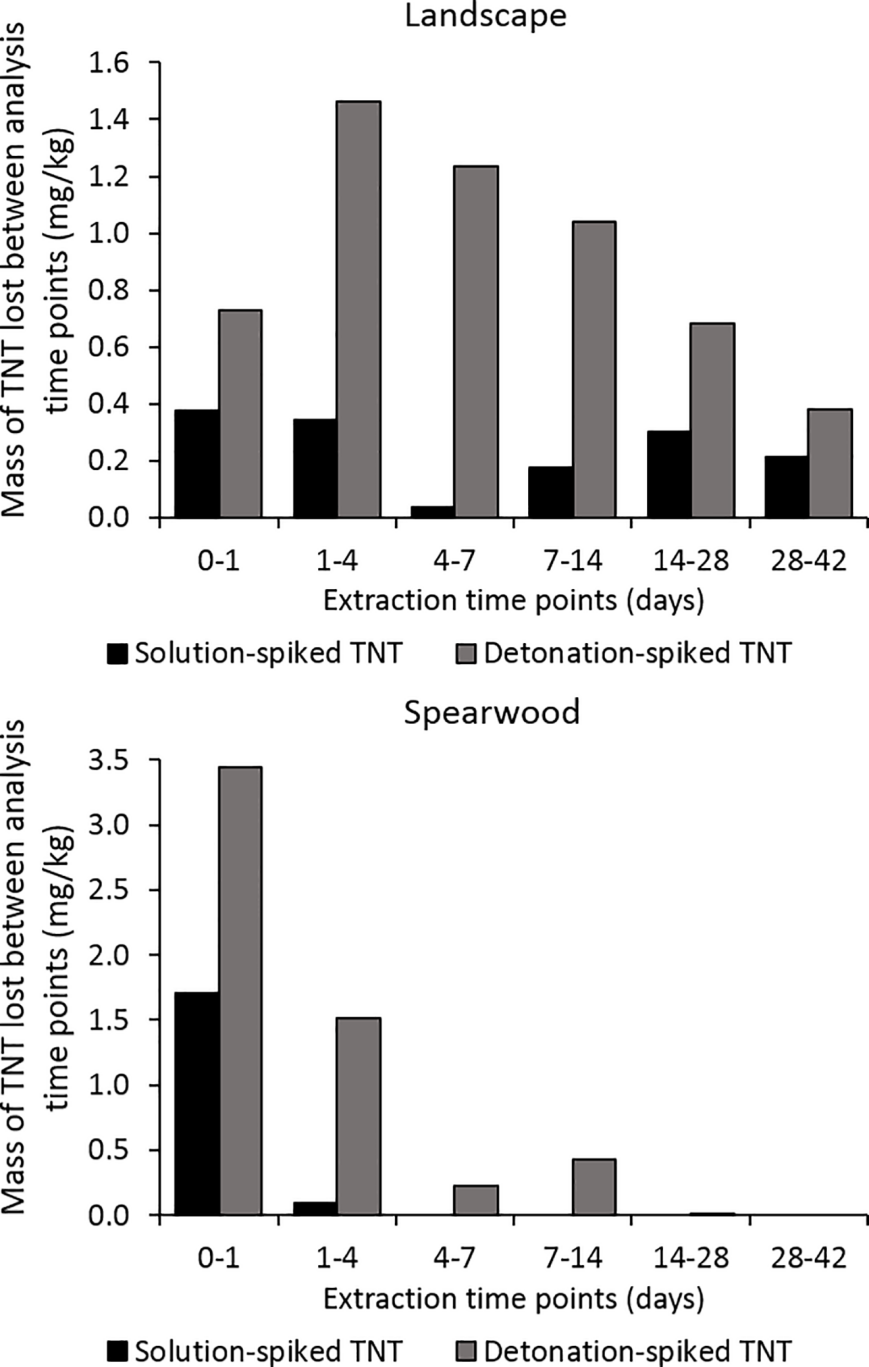
Average (n = 2) TNT loss between the analysis time points used for this work, from spiked pre-blast and post-blast samples.

Instead, using the average TNT loss in mg/kg between each of the respective analysis time points used throughout this study will reveal whether the rate of TNT loss differed significantly between the solution-spiked and detonated soils. From Fig 7 it can be seen that a much faster rate of TNT loss occurred from the detonated soils between each set of analysis time points, compared to the solution-spiked soils. It should be noted that two outlying results were removed from the Spearwood detonation-spiked results prior to this calculation–these are attributed to the presence of large, discrete TNT crystals within the soil samples which would have otherwise skewed the results. We observed high RSDs between the levels of TNT detected in a set of 6 samples used to assess TNT homogeneity within the post-blast Spearwood sand, reinforcing the likelihood that these outliers were due to heterogeneously-distributed TNT crystals.

As we have shown, the internal structure of these two soils is very different, with the presence of many pores within the landscape soil, but almost no pores observed within the Spearwood sand (Fig 2). Here, we propose that the pores within the landscape soil act as a ‘sink’ for the TNT, with the TNT migrating into the pores of the landscape soil and thus becoming less accessible for microbial degradation, leading to a resultant longer TNT half-life in the landscape soil.

This hypothesis stems from the findings of Akbari et al. [44], outlined earlier, who, when studying hexadecane biodegradation in soils, suggested that hexadecane undergoes initial binding onto a soil’s mineral surfaces, followed by movement of the hexadecane deeper into the soil aggregate’s structure.

Depending on the pore size in which the hexadecane settled, this may be too small to be accessed by bacteria, meaning the hexadecane would be protected from biodegradation. The authors suggest that for soils whose aggregates have a high proportion of small (<4 μm) pores, bioaccessibility of any contained compounds to bacteria will be limited, and thus a low degree of degradation may be observed. Conversely, for soils whose aggregates have larger pores, a higher degree of bioaccessibility is expected. However, the authors also propose that for coarse grained soils of a lower internal porosity (such as fine sands and gravel), the bioremediation endpoint of any contained compounds is likely to be dictated by the rate of biodegradation of the compound itself (if a soil has a negligible porosity, only a negligible proportion of the compound of interest is likely to be contained within the pores).

Therefore, in parallel with the arguments of Akbari et al. [44], a similar hypothesis may be drawn between the TNT and soils used within this work–it is likely that the TNT spiked onto the soils will have undergone some migration into the pores in the soil aggregates, making a proportion of the TNT inaccessible to the soil bacteria. This may explain why the rate of TNT loss from the solution-spiked landscape soil occurred more slowly across the 6-week experimental timeframe than from the Spearwood sand, as the landscape soil has a much more porous internal structure than the Spearwood sand (Fig 2). In contrast, as the Spearwood sand has very few fine pores within its grain structure, any TNT spiked into the soils would have remained on the surface of the soil particles and would have been fully exposed to the microbes within the soil, making it susceptible to very rapid microbial degradation.

A much faster rate of TNT loss was observed from the post-blast soils compared to the solution-spiked soils, and this coincided with an increase in the soil aggregates’ porosity in the post-blast soils (Fig 4). These results suggest a detonation-induced phenomenon is responsible for such a significant increase in the rate of TNT loss from the post-blast soils. We propose that the increase in porosity we observed in the post-blast soils facilitated bacterial movement through the soil aggregates, making it easier for the bacteria to access the TNT and bring about its transformation. In addition, our SEM images showed that the detonations gave rise to newly fractured exposed surfaces within the soils, and it has been reported [32] that fresh mineral surfaces may be more geochemically active than weathered surfaces, with organic compounds displaying a greater affinity for such freshly cleaved surfaces. For example, TNT is reported to form electron donor-acceptor complexes with the siloxane groups of minerals in soils [8, 22, 35]. For this reason, an enhanced rate of TNT adsorption and transformation is likely to have occurred on these freshly exposed surfaces [8].

We stored our samples at three different temperatures: room temperature, refrigerated (1°C) and frozen (-20°C), with TNT recoveries consistently higher over time from the refrigerated and frozen soils than those stored at room temperature (Fig 6). In contaminated soils within the environment, the geographic location of the soil may therefore have an influence on the rate of TNT loss. In a cooler climate or an area with limited direct sunlight, our results suggest that TNT loss would occur much more slowly, and remediation may be more difficult. In contrast, we would expect TNT loss to occur more rapidly in warmer countries.

**Fig 8:**
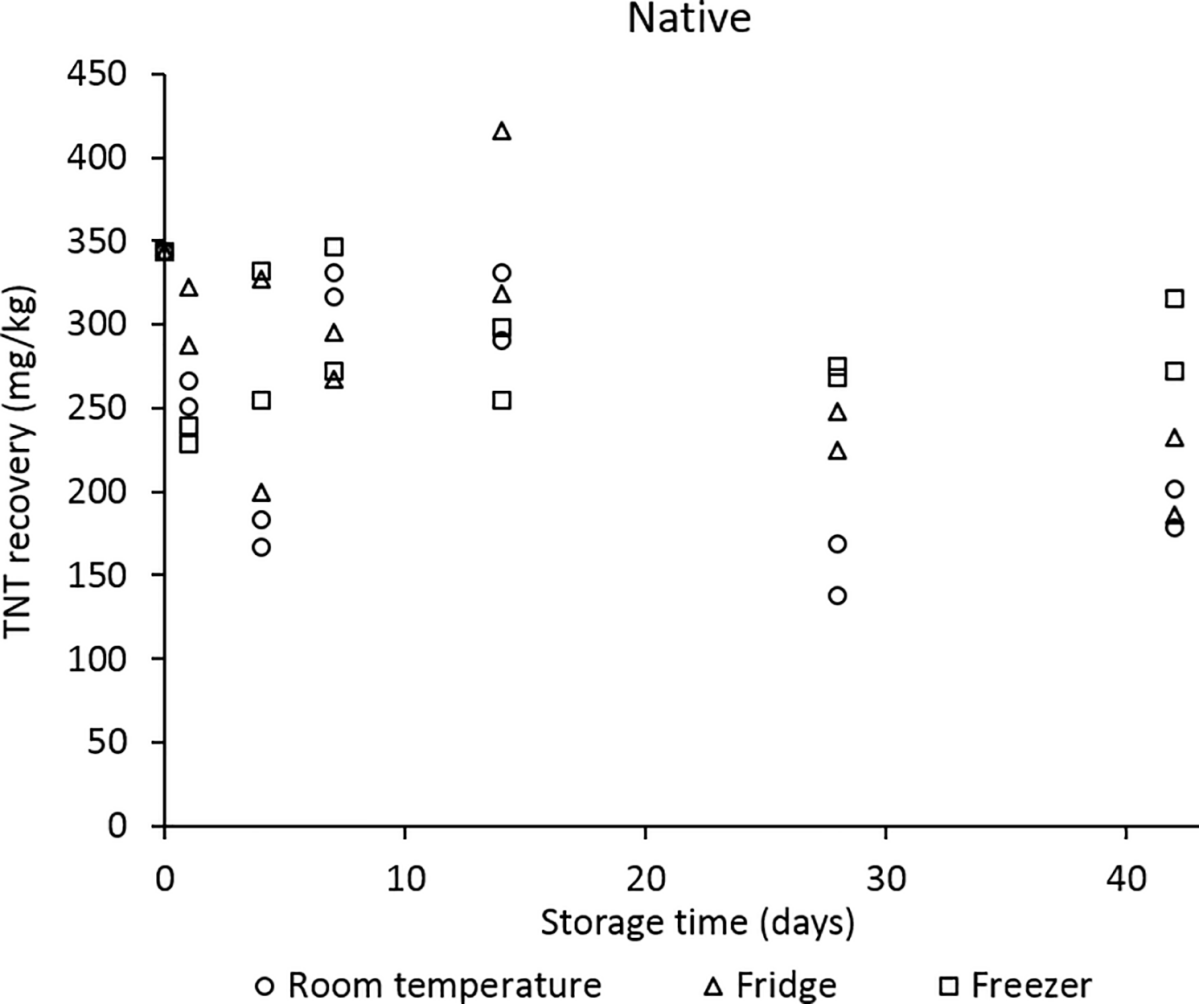
TNT recoveries obtained from post-blast native soil samples stored at 3 temperatures, extracting over 6 weeks.

All of the discussion up to this point has centred around TNT deposition into soils following an efficient explosion. However, during one of our detonations, a misfire occurred—the device did not detonate with ideal efficiency and much higher quantities of TNT were spiked into our native soil. In this case, even in the native soil samples stored at room temperature, we still observed high levels of TNT after 6 weeks (Fig 8)–in contrast to the results from the landscape and Spearwood soils, where the TNT recovery level fell substantially over the 6 weeks (Fig 6). Fig 8 displays the analogous TNT recoveries from post-blast native soil samples, extracting over 6 weeks.

**Fig 9:**
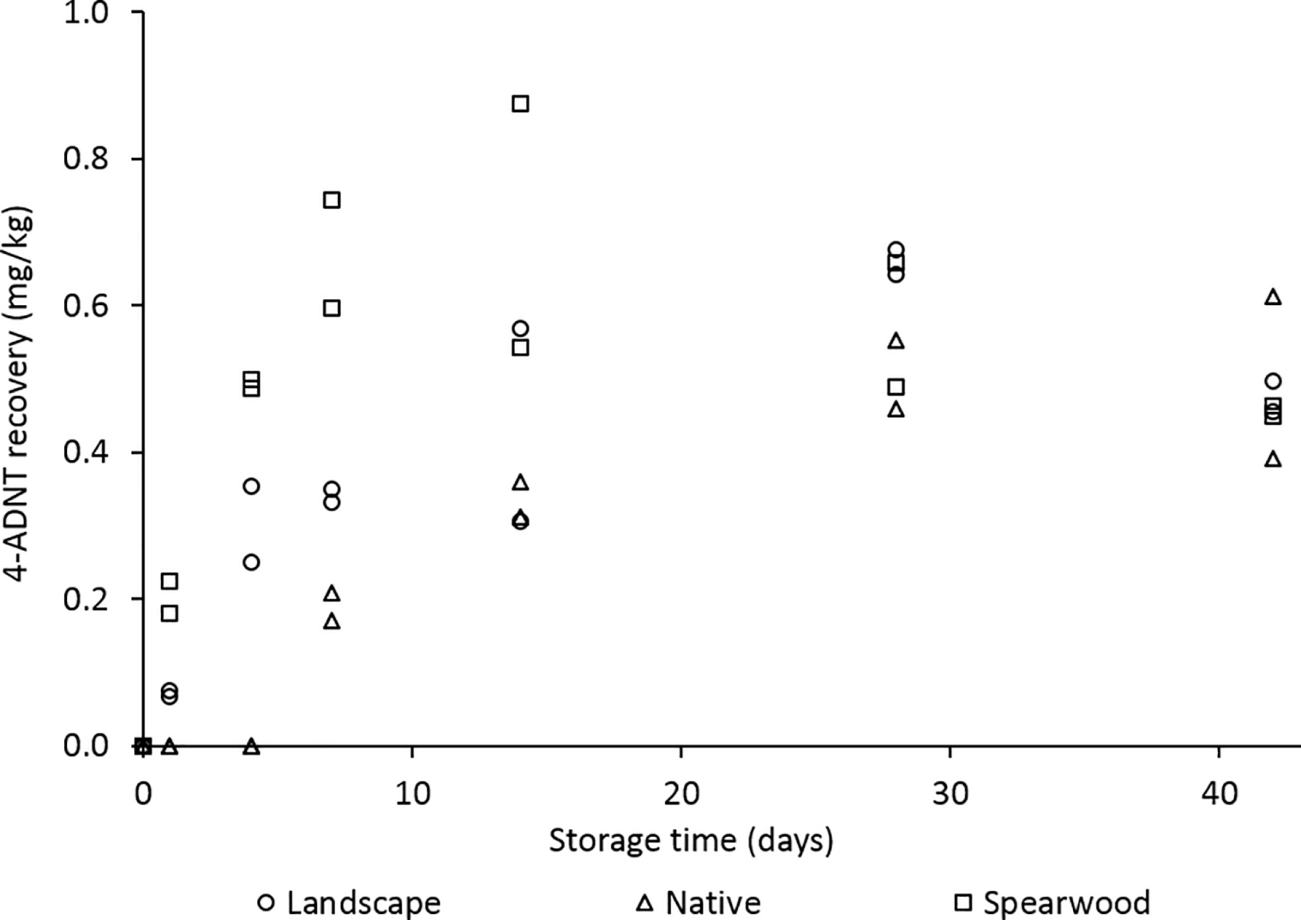
4-ADNT recovery from controlled detonation-spiked landscape, native and Spearwood soils stored at room temperature and extracted over 6 weeks.

Finally, Fig 9 displays recoveries of 4-ADNT from the three post-blast soils, stored at room temperature and extracted over 6 weeks.

In our native soil samples we observed an interesting effect with regards to the rate of formation of the major TNT microbial degradation product (4-ADNT)–with such high initial levels of TNT within the native soil samples (approximately 60x higher than in the detonation-spiked landscape and Spearwood soils), it may have been expected that this would lead to a corresponding 60-fold increase in the rate of TNT transformation and thus an enhanced rate of formation of the major TNT reduction product, 4-ADNT. However, this was not the case, and it was not until day 7 that we saw any 4-ADNT recovery (Fig 9) in the native soil, whereas 4-ADNT was recovered from the landscape and Spearwood samples after just 1 day. We propose that this is due to the high levels of TNT having an inhibitory effect on any TNT-transforming bacteria within the native soil, and that this time lapse is due to the bacteria adjusting to their heavily TNT-contaminated environment before they could begin to start transforming the TNT. Singh [7] has shown that high levels of TNT may have a toxic effect on bacteria in soils, and it is likely that this is the case in the native soil, accounting for the lower rate of observed microbial degradation. Other work [5] has also reported a prolonged TNT half-life due to high levels of TNT in soil and related bacterial inhibition processes.

## Conclusions

In this work, we assessed the effect of a detonation on soil structure, finding that detonations cause a shift to a smaller soil particle size, result in newly-fractured exposed grain surfaces and an increase in the porosity of soil aggregates.

Ultimately, the results from this work suggest that pores present within some soil types may act as ‘sinks’ for soil contaminants such as TNT, reducing its availability for subsequent microbial degradation and transformation. This has significant and far reaching implications for the remediation of explosives-contaminated sites. For example, if TNT runoff has occurred from contaminated land, then this TNT may build up and be stored within the surrounding soils, and the levels may remain high over time if bacteria are unable to access the TNT to bring about its transformation.

In addition to looking at the effect of a detonation on soil physical structure, we also assessed how the fate of TNT differed in pre-blast and post-blast soils, finding that TNT transformation occurred much more rapidly in post-blast soils than in our previously-reported solution-spiked soils [39]. In our post-blast soils, an increase in porosity was observed, and we propose that this increased rate of TNT transformation was the result of the TNT becoming more easily accessible for biodegradation to occur and to form less hazardous compounds such as 4-ADNT.

Our results have implications for the remediation of brownfield land for redevelopment and regeneration purposes. Based on the results of this work, in order to decontaminate or remediate sites with high levels of TNT, the porosity of the soils must be increased, potentially by crushing the soil or by performing a detonation in the vicinity of the soil (using a less persistent explosive, whose traces are not toxic or harmful to the environment), to break down the pore structure within the soil aggregates and release any contained TNT for transformation by bacteria present within the soils. TNT degradation and transformation in soils is brought about by non-specific NADPH-dependent nitroreductase enzymes, present in a large proportion of bacteria [10]. This means that if contaminated soil is treated following one of the pathways we suggest here, it is likely that a soil will contain a microbial community bearing this common enzyme, to catalyse the transformation of the TNT with a view to the regeneration and redevelopment of the land in question.

## Supporting information

S1 Table Properties of Spearwood sand, native and landscape soils used during this work (note that total organic carbon levels are determined independently from sand, silt and clay, so each row totals >100%). (XLSX)

S2 Table Individual and average aggregate porosities for the three soil types under the different conditions trialled. (XLSX)

S1 Fig Set up showing soil aggregates prepared for CT scanning, with (left) an aggregate mounted on the end of a pipette tip, and (right) an aggregate contained within a pipette tip. (TIF)

S2 Fig Size fraction distributions of coarser sized fractions for pre- and post-blast landscape, native and Spearwood soils. Error bars show standard deviations of three replicates.
(TIF)

S3 Fig SEM image of pre-blast landscape soil. Source: Evelyne Delbos, James Hutton Institute.
(TIF)

S4 Fig SEM image of pre-blast native soil. Source: Evelyne Delbos, James Hutton Institute.
(TIF)

S5 Fig SEM image of pre-blast Spearwood sand. Source: Evelyne Delbos, James Hutton Institute.
(TIF)

S1 File (DOCX)
