# Supplementary figures and images for: Explosive detonation causes an increase in soil porosity leading to increased TNT transformation
Source: PLoS One. 2017 Dec 27;12(12):e0189177. doi: 10.1371/journal.pone.0189177 (PMC5744939; doi:10.1371/journal.pone.0189177)

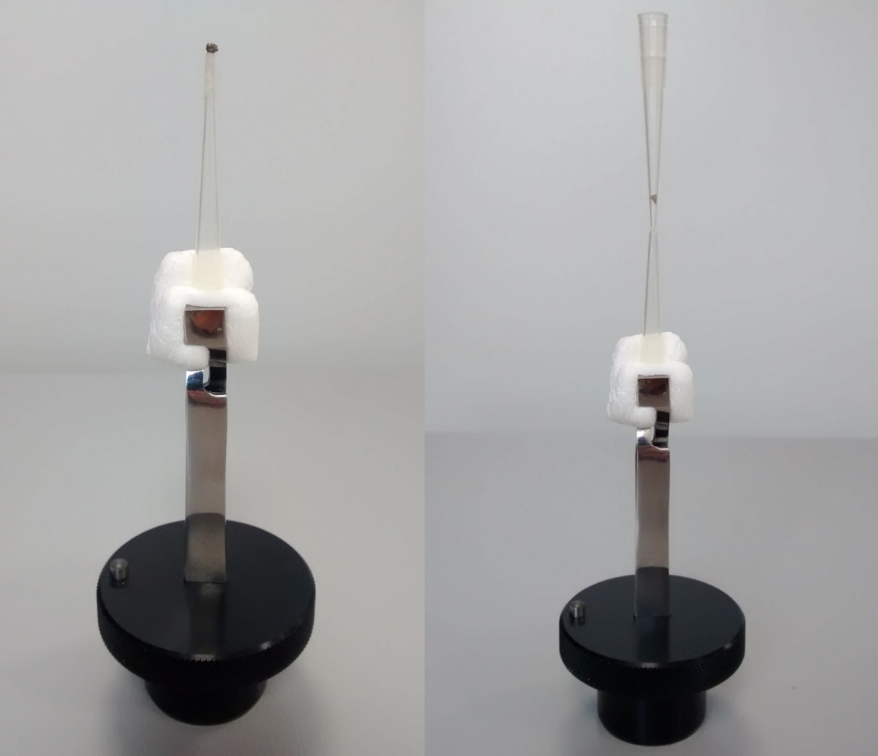

Supplement: S1 Fig — (TIF) [file pone.0189177.s003.tif]

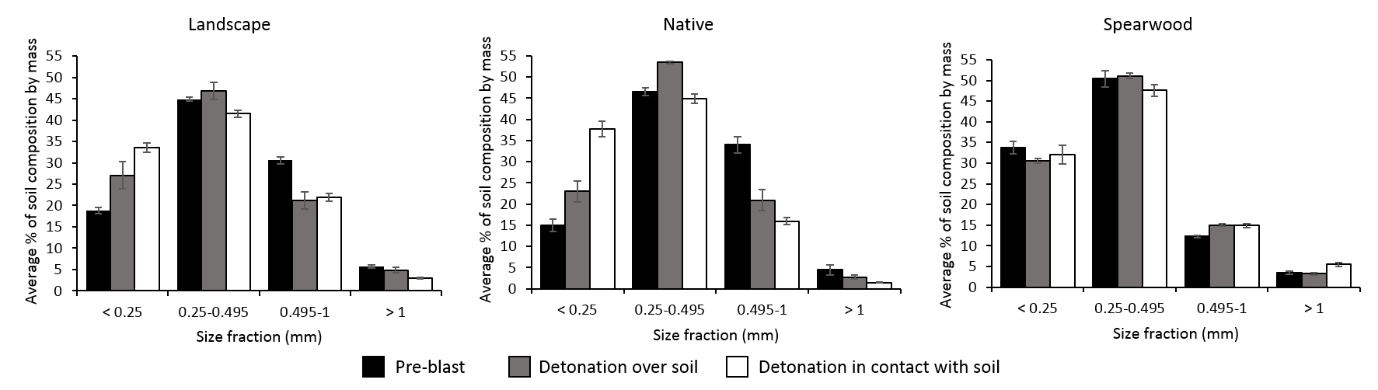

Supplement: S2 Fig — Error bars show standard deviations of three replicates. (TIF) [file pone.0189177.s004.tif]

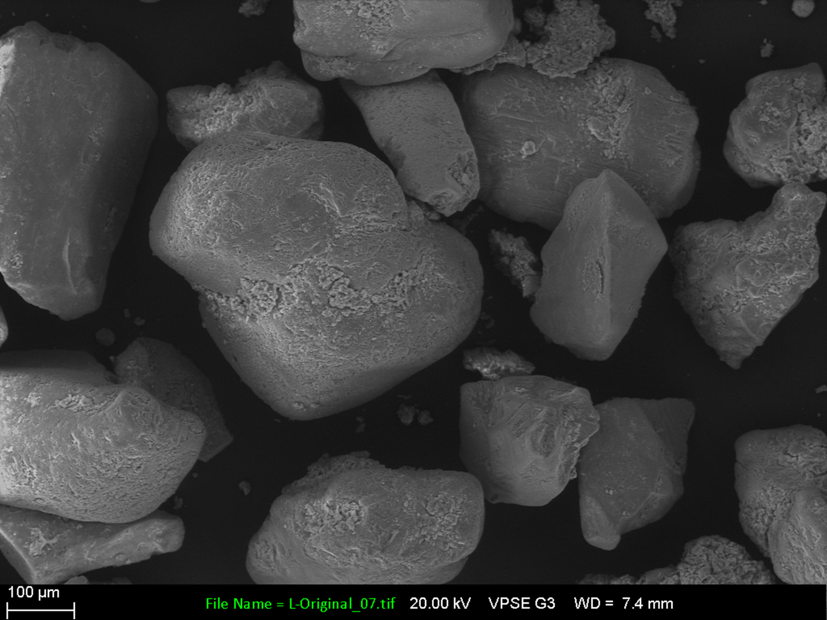

Supplement: S3 Fig — Source: Evelyne Delbos, James Hutton Institute. (TIF) [file pone.0189177.s005.tif]

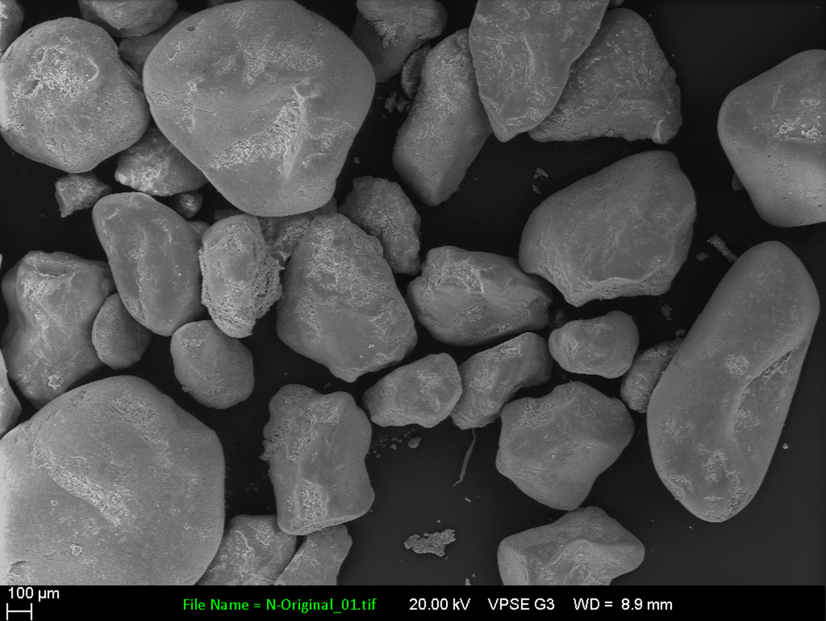

Supplement: S4 Fig — Source: Evelyne Delbos, James Hutton Institute. (TIF) [file pone.0189177.s006.tif]

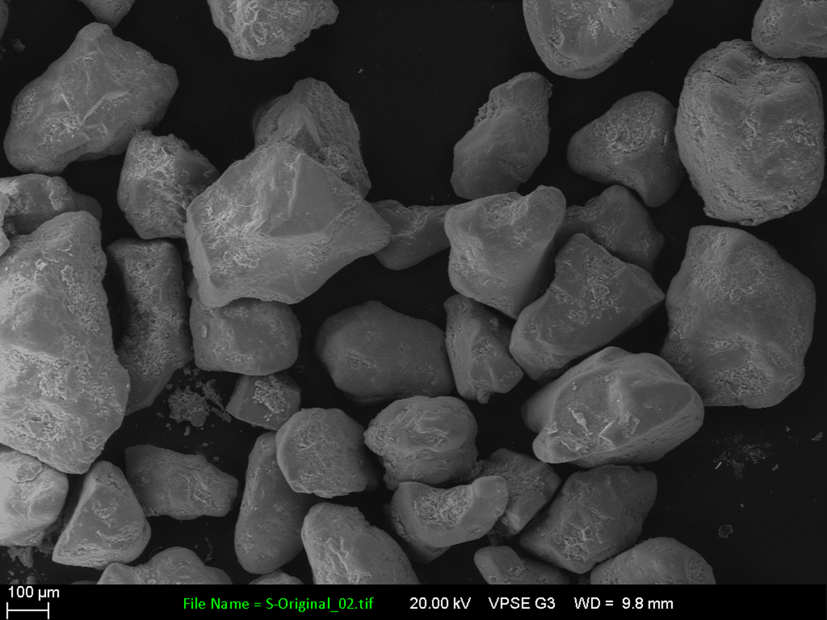

Supplement: S5 Fig — Source: Evelyne Delbos, James Hutton Institute. (TIF) [file pone.0189177.s007.tif]
