# Supplementary material for: Explosive detonation causes an increase in soil porosity leading to increased TNT transformation
Source: PLoS One. 2017 Dec 27;12(12):e0189177. doi: 10.1371/journal.pone.0189177 (PMC5744939; doi:10.1371/journal.pone.0189177)
Supplement: S1 File — (DOCX) [file pone.0189177.s008.docx]

**Supporting Information**

Explosive detonation causes an increase in soil porosity leading to increased TNT transformation

Holly A. Yu^1,2,3^, Niamh Nic Daeid^3*^, Lorna A. Dawson^4^, David A. DeTata^5^ and Simon W. Lewis^1,2^

^1^ Department of Chemistry, Curtin University, Perth, WA, Australia

^2^ Curtin Institute of Functional Molecules and Interfaces, Curtin University, Perth, WA, Australia

^3^ Centre for Anatomy and Human Identification, School of Science and Engineering, University of Dundee, Dundee, UK

^4^ The James Hutton Institute, Aberdeen, Scotland, UK

^5^ Forensic Science Laboratory, ChemCentre, Perth, WA, Australia

* Corresponding author

E-mail: n.nicdaeid@dundee.ac.uk (NND)

**Materials and Methods - Detonations and degradation experiments**

**Solvents and chemicals**

Acetonitrile (UV grade) was Honeywell, Burdick & Jackson Brand®. 18.2 MΩ cm^-1^ Milli-Q water was obtained from a Sartorius Arium 611VF water purification system. Explosives standards were obtained from LECO Australia, Castle Hill, NSW, and included the following: PETN, 1000 µg/mL in methanol; 2,4,6-Trinitrotoluene, 1000 µg/mL in acetonitrile; 2-amino-4,6-dinitrotoluene, 1000 µg/mL in acetonitrile; 4-amino-2,6-dinitrotoluene, 1000 µg/mL in acetonitrile; 1,2-dinitrobenzene (used as an internal standard), 1000 µg/mL in methanol.

**Consumables**

50 mL amber glass tall round bottles (24 mm) and white ringseal 24 mm polypropylene screwcaps were obtained from Cospak, Welshpool, Western Australia. 5 mL Luer slip syringes (without needle) were either Nipro Australia or Terumo brand. PTFE membrane 15 mm syringe filters (0.2 µm) were Phenomenex brand. The LC column used was an Agilent Zorbax Extend-C18 5 µm 4.6 x 150 mm column. LC vials were Agilent 2 mL clear screw cap vials or Phenomenex 2 mL clear Verex vials, 9 mm screw. 4.5 L HDPE plastic jars for storing wetted soil samples for the range day trials were obtained from Silverlock Packaging. Ezy Tarp 1.8 x 2.4 m tarpaulins with pegs were obtained from Bunnings, Western Australia. Grunt 200 µm black heavy duty builders’ film (2 x 20 m) was obtained from Bunnings, Western Australia. QiQ-Tidy 55 Litre metal bins were obtained from Bunnings, Western Australia. 25 g Pentex™ D Boosters and Orica Exel™ non-electric detonators were kindly supplied by the Western Australia Police Tactical Response Group – Bomb Response Unit.

**Soil preparation**

Spearwood sand soil was obtained from the Soil Laboratory of ChemCentre, Western Australia. Native and landscape soils were obtained from Soils Ain’t Soils, Perth, Western Australia. These three soils were chosen to offer a wide spread of different properties, to enable the behaviour of explosives to be assessed in a variety of different soils. The native soil was reported to contain sands, red loam and peat, whereas the landscape soil was reported to contain sands, fertilisers and peat. Analysis of each soil’s properties was performed by the Environmental Chemistry Section at ChemCentre. S1 Table contains selected information on the soil’s properties.

**S1 Table: Properties of Spearwood sand, native and landscape soils used during this work (note that total organic carbon levels are determined independently from sand, silt and clay, so each row totals >100 %)**

|  | Total organic carbon (%) | Sand (%) | Silt (%) | Clay (%) |
| --- | --- | --- | --- | --- |
| Spearwood sand | 0.95 | 95.5 | 1.5 | 3 |
| Native soil | 1.21 | 93.5 | 2.5 | 4 |
| Landscape soil | 1.76 | 92 | 3.5 | 4.5 |

All soils were dried in an oven at 40 °C for 48 hours and then sieved through a 2 mm sieve prior to use.

**High Performance Liquid Chromatography-Diode Array Detector (HPLC-DAD) Parameters**

Analyses were performed using an Agilent 1200 HPLC instrument equipped with a DAD detector. A flow rate of 1.6 mL/min was used, and a total run-time of 22 min. The following mobile phase sequence was used throughout this time: 0-11.5 min, 28:72 MeCN:H_2_O; 11.5-13 min, ramp to 50:50 MeCN:H_2_O; hold for 4 min; 17-18.5 min, decrease to 28:72 MeCN:H_2_O; hold for 3.5 min until end of run, with a constant column temperature of 40 °C used. TNT, 2-ADNT and 4-ADNT were detected at 240 nm, whereas 1,2-DNB and PETN were detected at 210 nm.

Calibration curves containing the analytes of interest (TNT, PETN, 2-ADNT and 4-ADNT) along with a suitable internal standard (1,2-DNB) were prepared in 52:48 MeCN:H_2_O to give on-column explosives levels of 5, 10, 20, 35, 50 and 100 ng, and 10 ng 1,2-DNB, following a 10 µL injection. The instrument was re-calibrated regularly, with the resulting calibration curves consistently giving R^2^ values of at least 0.998. Analysis was performed using ChemStation Rev. B.04.03-SP1 software.

**Materials and Methods - µCT analyses**

**Sample preparation**

Images of the two set ups used for µCT soil analyses are provided in S1 Fig.

**S1 Fig: Set up showing soil aggregates prepared for CT scanning, with (left) an aggregate mounted on the end of a pipette tip, and (right) an aggregate contained within a pipette tip**

**Image reconstruction**

Although the Inspect-X software associated with the CT scanner is capable of performing automatic reconstruction of the resulting image stacks, this tended to determine a less accurate centre of rotation, resulting in slightly blurred reconstructions. For this reason, reconstruction was performed separately from the scans, using Nikon Metrology X-TEK CT Pro 3D Version XT 4.3.1.

This involved opening the saved Extekct file and first comparing the ‘projection’ and ‘extra projection’ images (taken at the beginning and end of a scan, respectively), to ensure the sample had not moved significantly during the time taken to run the scan. Following this, the centre of rotation was determined via the centre of rotation tab, opting for the Automatic, High Accuracy and Dual Centre of Rotation options (N.B. in some cases, the dual centre of rotation option determined an incorrect axis of rotation, giving unsatisfactory images, so in these cases, the Single Centre of Rotation option was used instead).

Once a satisfactory centre of rotation had been achieved, the resulting data was sent for reconstruction in the Nikon Metrology X-TEK CT Agent program, Version XT 4.3.1, opting for a Floating Point volume, Full Range attenuation scaling and no volume graphics data import conversion. No beam hardening presets were used during the reconstruction.

Following reconstruction, the new VGL file was opened in VG StudioMax version 2.2.6, importing the volume in an unsigned 16 bit format. An image stack from one of the three available projections (Front, Top or Right) was then exported in Tiff format prior to further image processing using the ImageJ program.

**Thresholding**

The saved image stacks were imported into ImageJ version 1.50b. Prior to performing any porosity calculations, thresholding was performed. This was achieved using ImageJ’s Image 🡪 Adjust 🡪 Threshold option. The threshold value was determined for 5 slices within a stack (taking precedence from Pajor et al. (41)). In the current work, rather than opting for 5 random slices as reported by Pajor et al., representative slices from 10, 30, 50, 70 and 90 % of the way through the stack were used, manually adjusting the histogram if necessary to ensure that adequate delineation of the soil solid and pore space had been achieved. An average was then taken of these 5 threshold values and applied as an averaged global threshold across the entire image stack.

**Volume analysis and porosity determination**

Porosity percentages of the different aggregates were calculated using the ImageJ BoneJ plug-in (primarily designed for calculating bone density), version 1.4.1. The first step in this process was to ensure that a suitable threshold had been applied (see section above). Following this, suitable regions of interest (ROIs) had to be defined which would encompass the entire aggregate volume. For this, 30 regions of interest were defined in each image stack, approximately every 17 slices, using the ‘Create Selection’ feature. This feature selected all detected edges present within an image (including the edges of pores entirely contained within the centre of an aggregate). In order to calculate overall porosity, solely the edges of the aggregate needed selecting, rather than any edges present within an aggregate. For this reason, any selected internal edges were manually removed from the selection using the ‘Selection Brush’ tool. An example of an aggregate slice progressing through these stages (original aggregate slice, thresholded slice, and selection before and after its internal edges were removed) is displayed in Fig 3.

At this stage, the modified selection was added into the ROI manager. This process was repeated across 30 evenly-spaced slices through the aggregate, with each new selection added into the ROI manager. The ‘Interpolate’ feature within the ROI manager was then used, to generate an ROI on each individual slice.

At this stage, the Volume Fraction option from the BoneJ plug-in was used, to calculate the porosity within the defined regions of interest. This was performed using the Surface algorithm, with a surface resampling value of 6. It was ensured that the ‘Use ROI manager’ box was ticked, to ensure that the porosities were calculated for the pre-defined regions of interest. This generated a value designated BV/TV (arbitrarily referring to Bone Volume/Total Volume), indicating the proportion of solid material within the defined volume of interest (i.e. a value of 0.8 would indicate a volume of interest was 80 % solid and 20 % air).

**Results and discussion**

**Soil size fraction distributions**

S2 Fig displays the size fraction distributions of the three soils before and after detonation.

**S2 Fig: Size fraction distributions of coarser sized fractions for pre- and post-blast landscape, native and Spearwood soils. Error bars show standard deviations of three replicates.**

**SEM analyses of soils**

SEM images of pre-blast landscape, native and Spearwood soils are provided in S3-S5 Figs.

**S3 Fig: SEM image of pre-blast landscape soil. Source: Evelyne Delbos, James Hutton Institute**

**S4 Fig: SEM image of pre-blast native soil. Source: Evelyne Delbos, James Hutton Institute**

**S5 Fig: SEM image of pre-blast Spearwood sand. Source: Evelyne Delbos, James Hutton Institute**

**Volume analysis and porosity determination**

S2 Table displays the individual and average calculated porosities for the three soils exposed to the two detonation setups.

**S2 Table: Individual and average aggregate porosities for the three soil types under the different conditions trialled**

| Soil | Aggregate | Original soil - aggregate porosity (%) | Charge detonated over soil - aggregate porosity (%) | Charge detonated in contact with soil - aggregate porosity (%) |
| --- | --- | --- | --- | --- |
| Landscape | 1 | 14.7 | 8.8 | 16.9 |
|  | 2 | 5.4 | 14.8 | 14.9 |
|  | 3 | 7.5 | 22.2 | 13.0 |
|  | **Average** | **9.2** | **15.3** | **14.9** |
| Native | 1 | 11.0 | 11.1 | 16.2 |
|  | 2 | 9.2 | 14.7 | 17.6 |
|  | 3 | 3.6 | 8.7 | 6.2 |
|  | **Average** | **7.9** | **11.5** | **13.3** |
| Spearwood | 1 | 0.6 | 0.6 | 0.7 |
|  | 2 | 0.5 | 1.8 | 1.4 |
|  | 3 | 0.6 | 0.8 | 0.7 |
|  | **Average** | **0.6** | **1.1** | **0.9** |
